# Radiological Patterns of Pediatric Non-cystic Fibrosis Bronchiectasis: A Retrospective Study From Oman

**DOI:** 10.7759/cureus.86516

**Published:** 2025-06-22

**Authors:** Majid Al Jabri, Ons Al Humaimi, Fatma Al Yousufi, Abdullah Al Farsi, Tabinda Qureshi, Hussein Al Kindy

**Affiliations:** 1 Child Health Department, Sultan Qaboos University Hospital, Muscat, OMN; 2 Child Health Department, Sultan Qaboos University Hospital, College of Medicine and Health Sciences, Muscat, OMN

**Keywords:** ct imaging, non-cystic fibrosis bronchiectasis, primary ciliary dyskinesia, primary immunodeficiency, radiological features, sultan qaboos university hospital

## Abstract

Background: Non-cystic fibrosis bronchiectasis (NCFB) remains underdiagnosed in pediatric populations, particularly in the Middle East.

Objective: To characterize the high-resolution computed tomography (HRCT) features of NCFB in pediatric patients, including extent, morphological subtype, and lobar distribution, and to evaluate their associations with underlying clinical diagnoses at a tertiary care center in Oman.

Methods: We conducted a retrospective cross-sectional study at Sultan Qaboos University Hospital (SQUH), a tertiary center in Oman, reviewing pediatric patients ≤18 years diagnosed with NCFB between January 2000 and December 2022. High-resolution computed tomography (HRCT) reports prepared by pediatric radiologists were reviewed. Data on clinical features, radiological patterns, lobar involvement, and etiologies were analyzed descriptively using IBM SPSS Statistics for Macintosh, Version 19.0 (IBM Corp., Armonk, NY).

Results: Of the 150 patients reviewed, 61 met the inclusion criteria. The mean age at diagnosis was 7.3 years, with 35 (57.4%) being male. Diffuse bronchiectasis was predominant, observed in 48 patients (78.7%), and involved more than two lobes in 40 cases (65.6%). The left lower lobe was the most frequently affected, seen in 13 patients (21.6%). Cylindrical bronchiectasis was present in all patients, while cystic in 25 patients (41.0%) and varicose in 19 patients (31.1%) forms were more common in those with systemic disorders, such as primary immunodeficiency (PID, 18 patients; 37.5%) and primary ciliary dyskinesia (PCD, 8 patients; 16.7%). Patients diagnosed at age ≥5 years had a significantly higher prevalence of diffuse disease.

Conclusion: HRCT is a crucial diagnostic tool for pediatric NCFB, particularly in children with recurrent infections or systemic comorbidities, such as PID or PCD. Early imaging may prevent irreversible damage and guide targeted treatment. Establishing national guidelines for pediatric chest CT utilization and incorporating multidisciplinary assessments may improve diagnostic timeliness and outcomes.

## Introduction

Bronchiectasis, a chronic and often debilitating lung disease, is characterized by irreversible dilation of the bronchi, leading to recurrent infections, chronic inflammation, and progressive respiratory decline [1].

While cystic fibrosis (CF) is a well-known cause of bronchiectasis, non-CF forms are increasingly recognized as a distinct clinical entity in children [2]. These cases may arise from various etiologies, including recurrent infections, immune deficiencies, structural airway abnormalities, and genetic disorders such as primary ciliary dyskinesia (PCD) [3]. The prevalence varies globally, with reported incidence ranging from 0.2 per 100,000 child-years in England to 3.7 per 100,000 child-years in New Zealand [4]. Despite growing recognition, pediatric non-cystic fibrosis bronchiectasis (NCFB) remains underdiagnosed in many regions, particularly in developing countries, due to limited awareness, variable clinical presentations, and restricted access to diagnostic tools [2].

High-resolution computed tomography (HRCT) is the gold standard for diagnosing bronchiectasis, offering detailed visualization of airway abnormalities [5]. Timely identification using HRCT can facilitate early therapeutic interventions, potentially prevent disease progression, and improve long-term outcomes. However, routine use in children requires a balanced approach considering radiation exposure, cost, and clinical benefit.

Characteristic CT findings of bronchiectasis include bronchial dilation relative to adjacent pulmonary arteries (commonly referred to as the “signet ring” sign), an increased broncho-arterial ratio (typically greater than 1), and the absence of normal tapering of the airways. Additionally, dilated bronchi extending to the lung periphery are frequently observed [6].

In Oman and across the Middle East, there is a paucity of data on the prevalence, radiological patterns, and clinical correlates of pediatric NCFB. Understanding these factors is essential for developing context-specific diagnostic strategies and clinical guidelines.

Given the limited data on pediatric NCFB in Oman, this study aimed to examine its clinical characteristics and underlying etiologies in the affected population, as well as to characterize the HRCT findings, focusing on the extent, morphological patterns, and lobar involvement, and explore their relationship with the underlying causes.

## Materials and methods

This retrospective, single-center cross-sectional study included children aged 18 years or younger diagnosed with NCFB at Sultan Qaboos University Hospital (SQUH), Muscat, between January 2000 and December 2022, confirmed through clinical history and HRCT imaging. This study was approved by the institute’s research ethics committee from the College of Medicine and Health Sciences at Sultan Qaboos University on September 15, 2022 (MREC #2884). All medical records reviewed in this study were de-identified before analysis to ensure patient confidentiality.

Out of 150 pediatric cases initially suspected to have bronchiectasis, 30 were excluded due to confirmed cystic fibrosis, and 59 additional cases were excluded due to incomplete data, misclassification, or missing HRCT imaging, resulting in a final cohort of 61 patients who met the inclusion criteria.

Data were collected from the hospital’s information system for all potentially eligible patients. Each record was de-identified and reviewed independently by two authors for accuracy and completeness. Basic demographic and clinical details were recorded, including gender, age, symptom onset, age at diagnosis, underlying etiology, and presenting features at the time of diagnosis. Radiological assessments were reviewed using chest CT imaging reports retrieved from the Tract Care System, focusing on characteristic features of this chronic airway disease.

Diagnosis was confirmed based on standard imaging criteria. Bronchial dilation was identified when bronchi appeared larger than their accompanying pulmonary arteries, often measured using the broncho-arterial (BA) ratio, with a value greater than 1 considered diagnostic. Bronchial wall thickening was defined as an increased thickness of the bronchial wall relative to adjacent blood vessels, indicating chronic inflammation or fibrosis. Lack of bronchial tapering was diagnosed when bronchi failed to show normal narrowing over a 2 cm segment after branching, either in the same plane or on consecutive slices. Normal bronchi exhibit smooth, progressive tapering, whereas dilated bronchi may maintain or even increase in diameter toward the periphery. Air trapping was noted when areas of the lung appeared hyperlucent on expiratory CT images due to retained air, suggesting small airway obstruction. At thin-section CT, normal airways are typically not visualized within 1 cm of the pleural surface; therefore, the presence of visible bronchioles in this location is considered abnormal and supportive of bronchiectasis.

CT findings were further categorized into morphological subtypes. Cylindrical when the bronchial lumen had a smooth tubular outline, varicose when the lumen had a variable diameter, resulting in a beaded appearance, and cystic when the dilated bronchi appeared as rings or clusters of cysts.

The radiological changes were classified as focal if one to two lobes were involved unilaterally, or diffuse if two or more lobes were involved bilaterally.

Statistical analysis was performed using IBM SPSS Statistics for Macintosh, Version 19.0 (IBM Corp., Armonk, NY). Descriptive statistics were used to summarize clinical characteristics, radiological features, and etiological patterns. Frequencies and percentages were reported for categorical variables, and means with standard deviations were used for continuous variables.

## Results

A total of 61 pediatric patients with confirmed NCFB met the inclusion criteria and were included in the analysis. The majority were males (35/61, 57.4%), and the mean age at diagnosis was 7.3 ± 4.4 years.

Radiological patterns stratified by underlying etiology are detailed in Table 1, which summarizes the extent (focal vs. diffuse) and morphology (cylindrical, cystic, or varicose) of bronchiectasis. Cylindrical bronchiectasis was observed in all 61 patients (100.0%), while cystic bronchiectasis was present in 25 patients (41.0%) and varicose in 19 patients (31.1%). Among etiological groups, diffuse bronchiectasis was most common in those with primary immunodeficiency, 18/48 (37.5%), and idiopathic cases, 6/48 (12.5%).

**Table 1:** Radiological findings which show extent and type of bronchiectasis according to different diagnosis

Diagnosis	Extent of bronchiectasis		Type of bronchiectasis		
	Focal n (%)	Diffuse n (%)	Cylindrical n (%)	Cystic n (%)	Varicose n (%)
All the patients	13 (21.3)	48 (78.7)	61 (100.0)	25 (41.0)	19 (31.1)
Primary immunodeficiency	0 (0.0)	18 (37.5)	18 (29.5)	10 (40.0)	10 (52.6)
Primary ciliary dyskinesia	2 (15.4)	8 (16.7)	10 (16.4)	3 (12.0)	2 (10.5)
Severe infection	5 (38.5)	4 (8.3)	9 (14.8)	3 (12.0)	1 (5.3)
Idiopathic	0 (0.0)	6 (12.5)	6 (9.8)	5 (20.0)	3 (15.8)
Post infectious bronchiolitis obliterans	1 (7.7)	3 (6.3)	4 (6.6)	1 (4.0)	2 (10.5)
Post bone marrow transplant	0 (0.0)	3 (6.3)	3 (4.9)	1 (4.0)	0 (0.0)
Congenital lung disease	2 (15.4)	1 (2.1)	3 (4.9)	2 (8.0)	1 (5.3)
Chronic aspiration	1 (7.7)	1 (2.1)	2 (3.3)	0 (0.0)	0(0.0)
Leukemia	1 (7.7)	1 (2.1)	2 (3.3)	0 (0.0)	0(0.0)
Connective tissue diseases	1 (7.7)	1 (2.1)	2 (3.3)	0 (0.0)	0(0.0)
Others	0 (0.0)	2 (4.2)	2 (3.3)	0 (0.0)	0(0.0)

**Figure 1:**
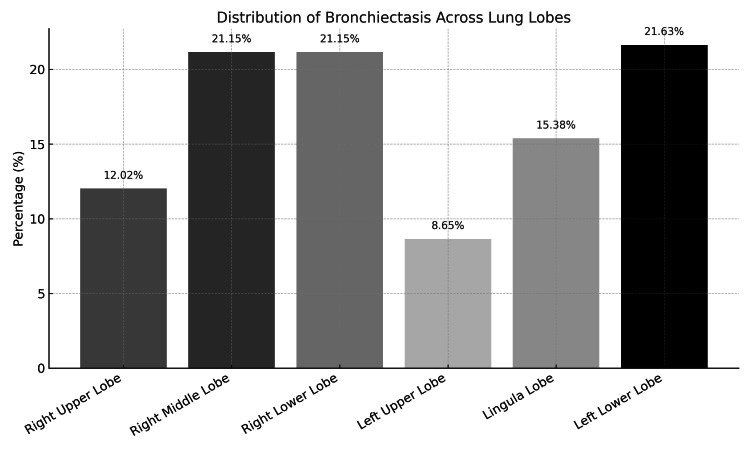
Distribution of bronchiectasis across lung lobes in the study population

As illustrated in Figure 1, the left lower lobe was the most frequently involved site 13/61 (21.6%), followed by the right lower lobe (21.1%) and the middle lobe (21.1%).

**Figure 2:**
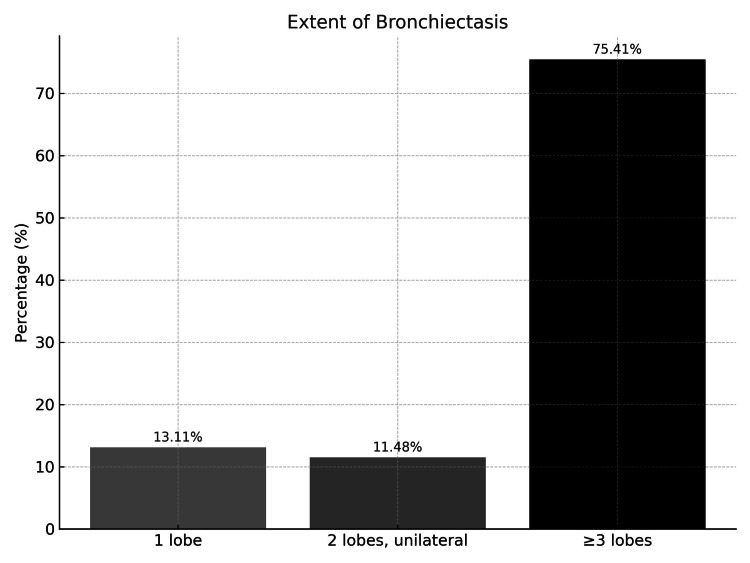
Extent of bronchiectasis at time of diagnosis

Furthermore, Figure 2 shows that more than two lobes were affected in 40 patients (40/61, 65.6%), indicating that diffuse and multilobar involvement was the predominant radiological pattern in this cohort.

HRCT findings showed that all 61 patients (100.0%) exhibited bronchial wall dilation, wall thickening, and a broncho-arterial ratio greater than one. Lack of bronchial tapering was observed in 28 (45.9%) and air trapping in 34 (56.7%) (Table 2).

**Table 2:** Frequency of bronchial abnormalities on HRCT HRCT: high-resolution computed tomography

Radiographic feature	N (%)
Bronchial wall dilation	61 (100.0%)
Bronchial wall thickness	61 (100.0%)
Lack of bronchial tapering	28 (45.9%)
Air trapping	34 (56.7%)
Motion or artifacts	5 (8.2%)

**Figure 3:**
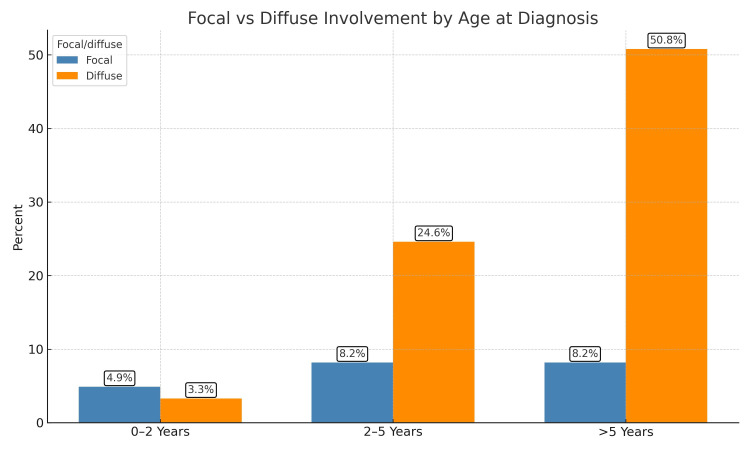
Relationship between age at diagnosis and extent of bronchiectasis

Among the 61 patients, those diagnosed at age ≥5 years had a significantly higher rate of diffuse bronchiectasis, 31 patients (50.8%), while children aged 0-2 years had a much lower incidence, with only two patients (3.3%) (Figure 3).

## Discussion

This study provides new insights into the radiological features and extent of NCFB. As the first report of its kind in the country, it contributes to the growing body of global literature on pediatric bronchiectasis and supports previous findings regarding disease presentation and progression in children. Our results align with prior studies from Turkey and Egypt, which similarly documented lower lobe predominance and multi-lobar involvement in pediatric NCFB cohorts [7,8].

NCFB is a heterogeneous condition with multiple contributing etiologies. A systematic review of nearly 1,000 pediatric cases found that infection accounted for 19%, primary immune deficiency 17%, aspiration/foreign body 10%, and primary ciliary dyskinesia (PCD) 7% of cases, while long-term bacterial bronchitis was also frequently described [3]. Our cohort reflected this diversity, with primary immunodeficiency and PCD being the leading causes, especially in patients with diffuse disease.

Lower lobe and right middle lobe involvement were most frequently observed, consistent with prior findings. One study reported that the lower lobes, particularly the left (71.1%) and right (59.4%), were most commonly involved, with bilateral disease in over 60% of cases [7]. In our cohort, the left and right lower lobes and the right middle lobe were affected in approximately 21% of patients each. These lobar predilections reflect impaired mucociliary clearance and recurrent infections, especially in children with PCD and immunodeficiencies [5].

More than two lobes were involved in the majority of cases (65.6%), consistent with diffuse disease patterns seen in children with systemic causes such as immunodeficiency and PCD. In contrast, cystic fibrosis (CF) typically affects the entire lung; however, the upper lobes are more frequently and severely involved. This is thought to be due to the prolonged stasis of thick, sticky mucus in these regions, leading to obstruction and infection [5]. Patients with focal bronchiectasis, on the other hand, were more likely to have antecedent severe infection or congenital airway anomalies. These findings underscore the value of detailed radiological evaluation in guiding further workup and individualized treatment strategies.

Radiological diagnosis of bronchiectasis relies on well-defined CT features. In our study, approximately half of the cohort demonstrated a lack of tapering, and 56.7% had evidence of air trapping, highlighting the chronicity and structural severity of the disease. Cystic and varicose morphologies were primarily associated with immunodeficiency and post-infectious etiologies, consistent with existing literature [9].

Age at diagnosis was found to influence disease extent. Diffuse bronchiectasis was markedly more common in children diagnosed at 5 years or older (50.8%), suggesting a longer subclinical course or delays in referral. This finding reinforces the need for earlier recognition and investigation, particularly in at-risk groups.

Limitations

Several limitations should be considered when interpreting these findings. Because SQUH is a national referral center, our cohort may overrepresent more severe cases, whereas milder disease may be managed in primary care settings. This referral bias could limit the generalizability of our results. Some referred patients had incomplete data, which limited the cohort size and the ability to perform subgroup analyses. Although we included a diverse range of etiologies, the relatively small sample size precluded meaningful statistical comparisons between subgroups. Additionally, while no radiologist was included among the authors, all radiological findings were based on formal reports issued by board-certified pediatric radiologists, ensuring diagnostic reliability. These limitations underscore the need for multicenter studies with larger cohorts.

Additionally, while no radiologist was included among the authors, all radiological findings were based on formal reports issued by board-certified pediatric radiologists, ensuring diagnostic reliability. These limitations underscore the need for multicenter studies with larger cohorts.

## Conclusions

HRCT remains an indispensable tool in the diagnosis and management of NCFB. Early screening and a high index of suspicion are critical for improving outcomes. While this study offers valuable national insights, its implications resonate globally and support the broader need for standardized diagnostic protocols and multicenter collaborations.
